# Fate of fluoroquinolones in field soil environment after incorporation of poultry litter from a farm with enrofloxacin administration via drinking water

**DOI:** 10.1007/s11356-024-32492-x

**Published:** 2024-02-17

**Authors:** Jan Fučík, Anna Amrichová, Kristýna Brabcová, Renata Karpíšková, Ivana Koláčková, Lucie Pokludová, Šárka Poláková, Ludmila Mravcová

**Affiliations:** 1 Institute of Environmental Chemistry, Faculty of Chemistry, Brno University of Technology, Purkyňova 118, 612 00 Brno, Czech Republic; 2 Department of Public Health, Faculty of Medicine, Masaryk University, Kamenice 5, 625 00 Brno, Czech Republic; 3 Institute for State Control of Veterinary Biologicals and Medicines (ISCVBM), Hudcova 56 A, Brno, Czech Republic; 4 Central Institute for Supervising and Testing in Agriculture (ÚKZÚZ), Hroznová 63/2, 603 00 Brno, Czech Republic

**Keywords:** Veterinary antimicrobials, Fluoroquinolones, Manure fertilization, Antimicrobial resistance, Solid phase extraction, Liquid chromatography, Mass spectrometry, PCR

## Abstract

The practice of incorporating animal manure into soil is supported within the European Circular economy as a possible substitute for mineral fertilizers and will become crucial for the sustainability of agriculture. However, this practice may indirectly contribute to the dissemination of antibiotics, resistance bacteria, and resistance genes. In this study, medicated drinking water and poultry litter samples were obtained from a broiler-chick farm. The obtained poultry litter was incorporated into the soil at the experimental field site. The objectives of this research project were first to develop analytical methods able to quantify fluoroquinolones (FQs) in medicated drinking water, poultry litter, and soil samples by LC–MS; second to study the fate of these FQs in the soil environment after incorporation of poultry litter from flock medicated by enrofloxacin (ENR); and third to screen the occurrence of selected fluoroquinolone resistance encoding genes in poultry litter and soil samples (PCR analysis). FQs were quantified in the broiler farm’s medicated drinking water (41.0 ± 0.3 mg∙L^−1^ of ENR) and poultry litter (up to 70 mg∙kg^−1^ of FQs). The persistence of FQs in the soil environment over 112 days was monitored and evaluated (ENR concentrations ranged from 36 μg∙kg^−1^ to 9 μg∙kg^−1^ after 100 days). The presence of resistance genes was confirmed in both poultry litter and soil samples, in agreement with the risk assessment for the selection of AMR in soil based on ENR concentrations. This work provides a new, comprehensive perspective on the entry and long-term fate of antimicrobials in the terrestrial environment and their consequences after the incorporation of poultry litter into agricultural fields.

**Graphical Abstract:**

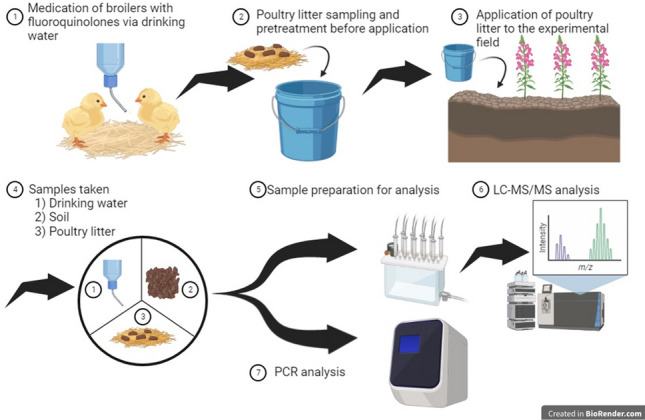

**Supplementary Information:**

The online version contains supplementary material available at 10.1007/s11356-024-32492-x.

## Introduction

The widespread use of antibiotics plays a key role in the management of infectious diseases in livestock (including poultry) (Cycoń et al. 2019; Pan and Chu 2017; Rakonjac et al. 2022; Schlüsener and Bester 2006; Solliec et al. 2016). Veterinary antibiotics (VAs) are not only used for therapeutic purposes but also used to increase production in animal husbandry in some non-European countries (the EU banned this practice in 2006). Any administration of antimicrobial, notwithstanding on fact if therapeutical or as an antimicrobial growth promoter (AGP), is linked with the excretion of it or its metabolites into the environment (up to 90% of the drug dosage) (Aga et al. 2016; Hu et al. 2010; Jechalke et al. 2014; Martinez et al. 2006; Pan and Chu 2017; Schlüsener and Bester 2006; Solliec et al. 2016).

For this study, enrofloxacin (ENR) was chosen as one of the most important FQ, which is often used to treat respiratory and digestive tract-affecting diseases of broiler-chicks. According to European Medicines Agency (EMA) reports on sales of veterinary antimicrobial agents, a total of 146.4 tonnes of fluoroquinolones (FQs) were sold in 2021 in 31 EU/EEA countries (stagnation of consumption since 2017). Approximately 60–87% of ENR is excreted non-metabolized and the rest is excreted mainly as ciprofloxacin (CIP) (Slana and Sollner-Dolenc 2015).

Subsequently, these compounds can be introduced into the soil environment via the incorporation of animal manure (Bartrons and Peñuelas 2017; Gros et al. 2019b; Ho et al. 2014; Kodešová et al. 2015; Pan and Chu 2016; Pan and Chu 2017; Pino et al. 2015; Rakonjac et al. 2022; Schlüsener and Bester 2006; Solliec et al. 2016). Poultry litter is considered to be the main source of FQs in agricultural fields (Han et al. 2018). Under general conditions, most VAs are degradable in soil, especially in the presence of manure, with DT_50_ (half-life) < 30 days (e.g. β-lactam antibiotics) (Pikkemaat et al. 2016). However, certain antibiotics such as macrolides and fluoroquinolones can persist for over 120 days (Song and Guo 2014). The degradation of pharmaceuticals in soil is a comprehensive result of microbial decomposition and environmental processes (Bartrons and Peñuelas 2017; Schlüsener and Bester 2006; Song and Guo 2014). The wide range of DT_50_ values shows that persistence depends on several different factors, e.g. physico-chemical properties of VAs, characteristics of the soil (e.g. organic matter content and pH) and climatic factors, such as temperature, rainfall, and humidity (Bartrons and Peñuelas 2017; Song and Guo 2014).

Recycling practice of animal manure presents multiple economic (waste management) and environmental benefits (Gros et al. 2019a; Pan and Chu 2017). Manure is commonly applied to agricultural crop fields as a nutrient-rich bio-based fertilizer (BBFs) (Gros et al. 2019a, 2019b; Jechalke et al. 2014; Kodešová et al. 2015). This practice is supported within the European Circular economy as a possible substitution for mineral fertilizers and will become crucial for the sustainability of agriculture given the growing population and lack of nutrients (Bartrons and Peñuelas 2017; Deschamps et al. 2014; Pan and Chu 2017). This circular economy may indirectly contribute to pharmaceuticals, resistant bacteria, and resistance genes dissemination into the environment (Deschamps et al. 2014; Jechalke et al. 2014). There are many functionally different mechanisms of bacterial resistance to fluoroquinolones. Some of them are chromosome-encoded, but more emphasis is placed on those mechanisms that are encoded by plasmid-carried genes. This is called plasmid-mediated quinolone resistance (PMQR), and due to the possibility that these genes could be transmitted horizontally as well as vertically, it is an emerging medical problem (Veldman et al. 2011).

The highest concentrations of pharmaceuticals (PhACs) are usually found in areas amended with manure, wastewater, or biosolids (Cycoń et al. 2019; Pan and Chu 2017). Several studies have shown that crops grown in amended soils can contain trace levels of pharmaceutical mixtures, which raises concerns over the potential human health risks associated with long-term dietary intake of PhACs. Contamination of soil with PhACs can also inhibit of seed germination and crop growth (Gros et al. 2019b; Ho et al. 2014; Kodešová et al. 2015; Kuppusamy et al. 2018; Li et al. 2020; Pan and Chu 2017; Rakonjac et al. 2022). According to statistical estimates by Murray et al. (2022), up to 1.17 million deaths worldwide are caused by antimicrobial resistance (AMR), and an additional 2.62 to 4.78 million deaths are associated with AMR.

Several environmental and monitoring studies dealing with the assessment of pharmaceutical contamination and fate in the agricultural field due to amendment by animal manure have already been conducted (Albero et al. 2018; Gros et al. 2019b; Qian et al. 2022; Quaik et al. 2020). Some studies have also investigated the presence of antimicrobial resistance genes (ARGs) in animal manure or the environment (Veldman et al. 2011; Xu et al. 2020). However, there is still a lack of studies investigating both concentrations of antimicrobials and ARGs within the same study and not only in the soil environment but also in medicated drinking water and poultry litter, before they enter the terrestrial environment. This is necessary to assess the whole environmental issue comprehensively from entry to the long-term fate of these antimicrobials in the soil and not only monitor their concentrations/consequences in the environment.

Hence, in the present work, medicated drinking water and contaminated poultry litter samples were obtained from broiler farms under common working conditions. This was followed by the incorporation of the contaminated poultry litter into the soil in our experimental field, where soil samples were obtained over a period of 112 days. First, analytical methods for the determination of fluoroquinolones in different matrices (medicated drinking water, poultry litter and soil) were developed. The presence of ENR and its metabolite CIP in naturally contaminated poultry litter samples and later in the soil samples was quantified via presented extraction methods and LC–MS/MS analysis. Furthermore, to assess environmental and health concerns of antimicrobials in the environment from multiple perspectives, PCR analysis of PMQR in poultry litter and soil samples was carried out. Additionally, analysis of the physico-chemical properties of the soil was carried out, and meteorological conditions were monitored during the experiment.

## Materials and methods

### Chemicals, solvents, and standards

Citric acid monohydrate (≥ 99%), disodium hydrogen phosphate dodecahydrate (≥ 99%), EDTA (Ethylendiaminetetraacetic acid; p.a.), sodium chloride (p.a.), magnesium sulfate anhydrous (p.a.), Sodium sulfate anhydrous (p.a.) and hydrochloric acid (35%) were purchased from Lach:ner (Czech Republic). Magnesium nitrate hexahydrate (> 99%), ammonium (25%), methanol (LC–MS grade), acetonitrile (LC–MS grade), and water (LC–MS grade) were purchased from VWR (USA). Sodium hydroxide (> 98%) was purchased from Penta. Formic acid (LC–MS grade), enrofloxacin (> 99%), ciprofloxacin (> 98%), enrofloxacin-d_5_ (> 99%), and ciprofloxacin-d_8_ (> 99%) were purchased from Sigma Aldrich (USA). OASIS HLB (Hydrophylic-Lipophilic Balanced) cartridges (200 mg; 6 mL; particle diameter 25–35 μm) were purchased from WATERS (USA). Nitrogen (4.7) and argon (5.0) gas were purchased from Siad (Czech Republic). Nylon syringe filters (13 mm, 0.22 μm) were purchased from Chromservis (Czech Republic). Physico-chemical properties of FQs can be found in Table S1.

### Description of broiler’s farm

The broiler-chicks’ farm sampled is located in the Czech Republic (Olomouc region) and has a capacity of one hall with up to 20,000 chickens. One-day-old chickens are brought to the farm and are already vaccinated against infectious bronchitis. In addition, on the day of arrival, a bacteriological examination is carried out on randomly selected chickens before they come into contact with the freshly prepared poultry litter, which consists of cardboard boxes during the first few days and later cardboard boxes with cut straw. For the first days, the houses are heated to 33 °C and flocks are usually exposed to 23 h of light and one hour of night light (usually blue light spectrum). Lightening regimen (together with other environmental parameters, e.g. temperature and humidity) is specifically set for broiler breed hybrids as one of the tools that helps to drive performance characteristics (including daily weight gain) as it influences intake of grain and water as well as activity of birds. During the growth process, the temperature is gradually reduced to the physiological/productive temperature of the chickens (the night lighting regime is also gradually extended). The growth process usually takes approximately 37–42 days.

Prophylaxis was indicated for the chickens (risk of *E. coli*, *Enterococcus faecium*, or *S. aureus* infections) based on lack of weight uniformity; number of dead birds at delivery; clinical/post mortem observations; experience of the attending veterinarian; and supported by bacteriological sampling/results from one-day old chicks at delivery. Therefore, the veterinary medicinal product ROXACIN in the form of a concentrate for the preparation of an oral solution containing enrofloxacin was administered to the chickens in medicated drinking water for 4 days. The daily dose was always prepared fresh, and the diluted concentrated solution (in 8–10 L of water) was subsequently added to the total expected volume of water consumed by the broilers at a dose equivalent to 10 mg/kg/day.

#### Sample collection at broiler’s farm

At the start of the medication by ROXACIN, a sample of drinking water (already medicated) was taken from the broilers’ drinking water device at the beginning of the pipeline in the hall and another from the end of the pipeline through poultry drinker. Sterilized, amber glass bottles (0.5 L) were used to collect water samples (*N* = 3); prior to the sample collection, the bottles were rinsed in triplicate with the water being sampled. Samples were transported back to the laboratory in cool boxes and stored at − 80 °C until analysis.

The initial poultry litter sample was collected shortly after bringing broilers to the farm, specifically on the 1st day of medication. Subsequent poultry litter samples were collected on the 3rd and 7th days of the medication. A systematic random sampling approach was employed, utilizing a square grid with 10-m intervals. Twelve subsamples were systematically collected from various locations across the entire poultry hall. To ensure a representative composite sample for each sampling day, the collected subsamples underwent a thorough homogenization process. This homogenization step aimed to eliminate potential spatial variations in litter composition within the hall, providing a more accurate representation of the overall conditions. Following homogenization, the uniform composite sample for each sampling day was placed in polyethylene (PE) bags and stored at − 80 °C until analysis.

### Field experiments

The experimental field was established on the premises of the ÚKZÚZ (Central Institute for Supervising and Testing in Agriculture) test station in Brno-Chrlice (Czech Republic). The field trial was conducted from May 2021 to September 2021. There were 2 plots in the trial, each of size 1 × 4 of meters, and Scorpion Weed (*Phacelia tanacetifolia*) was planted on the field.

Before the incorporation of poultry litter, a soil sample was taken and the physico-chemical properties of the soil were determined (Table S2 and Table S3). Furthermore, meteorological conditions were monitored during the experiment. According to the data from this station (Fig. S1), the range of temperatures from the start of the experiment (20 May 2021) to the end of the experiment (9 September 2021) was 0.1 °C to 34.6 °C. Rainfall totals ranged from 10.6 mm in September to 131.5 mm in August. Overall, monthly precipitation in the experimental field was slightly lower every month than the normal precipitation (average of the precipitation values over a 30-year period).

#### Incorporation of poultry litter to the soil

The homogenized poultry litter was uniformly incorporated into the experimental plot on 20 May 2021. Prior to incorporation, the poultry litter was carefully weighed (3.92 kg per one experimental plot of 4 m^2^). To facilitate thorough homogenization, the weighed poultry litter was diluted with 7 L of deionized water. The dilution process ensured optimal consistency for homogenization and subsequent incorporation in the experimental field. Homogenization was achieved through the use of a mixing machine, ensuring precision and consistency in the blend. The application rate was calculated to give a predicted nitrogen application rate of 200 kg∙ha^−1^. The resulting incorporation was 0.98 kg of poultry litter per 1 m^2^. Before poultry litter incorporation into the soil, the obtained poultry litter was left to mature for 4 weeks instead of the usual/recommended 3 months, as the aim was to achieve one of the possible worst-case scenarios. The experimental plot was divided into two parts: (A) natural poultry litter (without further enrofloxacin enrichment) and (B) spiked poultry litter (enriched with enrofloxacin), so that the soil contained about 100 µg∙kg^−1^ of ENR more than soil with non-spiked poultry litter. The dose of 30 mg ENR per 1 m^2^ of soil was calculated using Eq. 1.

1 $${m}_{{\text{ENR}}}={\Delta c}_{{\text{ENR}}}\bullet {m}_{{\text{soil}}}={\Delta c}_{{\text{ENR}}}\bullet {\rho }_{{\text{soil}}}\bullet {S}_{{\text{soil}}}\bullet {h}_{{\text{soil}}}$$

where *m*_ENR_ is the spiking weight of ENR for 1m^2^ of soil, Δ*c*_ENR_ is enrofloxacin spiking concentration difference in the soil, *ρ* is the soil density (1500 kg∙m^−3^ for silt loam soil) (Zeri et al. 2018), *S*_soil_ is the soil area, and *h* soil is the height of the soil surface Sect. (20 cm due to low mobility of FQs in the soil environment) (Yu et al. 2012).

##### Sampling of soil from experimental field

Soil samples were collected before, immediately after the incorporation of poultry litter, one week after incorporation, and then periodically every two weeks. A total of 21 soil samples were obtained from the two experimental fields. An Edelman auger was used to collect the soil samples. One soil sample was collected from each portion of the experimental plot, which consisted of 18 individual subsamples. The depth of sampling was 15 cm. The soil sample was sieved through a 5-mm mesh. The samples were placed in a PE bag and frozen at − 80 °C until analysis.

### Analysis of medicated drinking water

Medicated drinking water contains high concentrations of FQs due to the necessary therapeutic effect on broilers. These water samples were analysed via direct injection, without extensive sample preparation, non-diluted and 100 × diluted by 0.1% FA in H_2_O:ACN (95:5). Before LC–MS/MS analysis (section “LC–MS/MS method for quantification of FQs”), the samples were filtered through 0.22-μm nylon syringe filters (diameter 13 mm).

The method validation results for medicated drinking water samples are shown in Table 1. For the quantification of FQs in medicated drinking water, external calibration was used. The calibration range was 0.1 to 500 ng∙mL^−1^ for both FQs. The coefficients of determination (*R*^2^) for ENR and CIP were > 0.995. The method limit of detection (LoD) and limit of quantification (LoQ) were calculated according to Eqs. 2–3 (method based on the standard deviation of the response and the slope). Intra-day precision and inter-day precision of the method were calculated in terms of RSD% of the QC samples (Eq. 4); however, accuracy and precision were also checked by analysis of the QC samples within each LC–MS run. The selectivity/specificity of the method was assessed by analysing of water blank samples. Confirmation of identity was achieved by monitoring the ion ratio of multiple MS/MS transitions in the MRM mode during LC–MS/MS analysis.

2 $${\text{LoD}}=3.3\bullet \frac{\sigma }{S}$$

where *σ* stands for the standard deviation of the blank response and *S* is the slope of the calibration curve.

3 $${\text{LoQ}}=10\bullet \frac{\sigma }{S}$$

where *σ* stands for the standard deviation of the blank response and *S* is the slope of the calibration curve.

**Table 1:** Method validation for the medicated drinking water samples

Analyte name	LoD (ng∙mL^−1^)	LoQ (ng∙mL^−1^)	RSD%
ENR	0.35	1.91	8.2
CIP	0.63	1.91	10.4

4 $$\mathrm{RSD \%}=\frac{\mathrm{Standard deviation of QC samples}}{\mathrm{Mean QC samples}}\bullet 100$$

### Extraction of fluoroquinolones from poultry litter and soil samples

All poultry litter and soil samples were placed in a hood and air-dried at laboratory temperature until a constant weight was obtained. Consequently, poultry litter was separated into 2 different subsamples: (A) only faeces and (B) whole poultry litter sample. Dried poultry litter was cut into small pieces and completely homogenized using a blender.

For the extraction of FQs from poultry litter and soil samples, the same ultrasound-assisted extraction (UAE) method was used. With the difference, that after the extraction, poultry litter extracts were analysed without further sample preparation, as they contain high concentrations of FQs and soil extracts were further processed using SPE, because the concentrations of FQs in soil are expected to be lower in comparison with water and poultry litter samples. In this case, this phenomenon is caused by soil dilution (An et al. 2015). In addition, the soil matrix is much more complex than animal manure or medicated drinking water.

To extract FQs from soil or poultry litter samples: 1 g of homogenized soil (or poultry litter) was weighed in a 50-mL PE centrifugation tube. The extraction was carried out following the procedure: 0.6 g of Na_2_EDTA, 7.5 mL of McIlvane buffer (pH 6.0), 7.5 mL of acetonitrile (ACN), 4.8 mL of Mg(NO_3_)_2_∙6H_2_O aqueous solution (concentration 0.5 g∙mL^−1^), and 0.2 mL of 2.5% NH_3_ aqueous solution were added to centrifugation tube (composition of extraction media is optimized parameter as described in the “Influence of the composition of different extraction media on recovery rate” section); followed by shaking by vortex for 30 s. After that FQs were extracted immediately by ultrasound bath for 10 min (temperature 35 °C; optimized parameter as described in the “Influence of extraction temperature in ultrasound bath on recovery rate” section). Sonication was followed by centrifugation (4800 rpm) for 8 min. After that, the supernatant was put in a glass vial of volume 50 mL. The second extraction run was then repeated with half the volume of extraction medium without the addition of Na_2_EDTA (3.75 mL of McIlvane buffer; 3.75 mL of ACN; 2.4 mL of Mg(NO_3_)_2_ solution and 0.1 mL of 2.5% NH_3_). Finally, both supernatants are placed in a glass vial.

In the case of poultry litter, extracts were analysed non-diluted or 10x-diluted by 0.1% FA in H_2_O:ACN (95:5). Subsequently to 1 mL of sample, 10 μL of a mixture of internal standards (mixture concentration 10 μg∙mL^−1^) was added. Before LC–MS/MS analysis, the poultry litter samples were filtered through 0.22-μm nylon syringe filters (diameter 13 mm). Subsequently, FQ concentrations in poultry litter samples were calculated according to Eq. 5.

5 $$\mathrm{FQs in poultry litter}=\frac{\mathrm{Measured analyte concentration }\bullet {V}_{\mathrm{total of extract }} \bullet {F}_{{\text{dilution}}} }{\mathrm{Sample weight}}$$

where *V*_total of extract_ is the total volume used for extraction (30 mL) and *F*_dilution_ is the factor of dillution used to dilute the sample before IS spiking, sample filtration, and analysis (0 or 10).

Meanwhile, soil extracts were diluted before SPE by adding 480 mL of Milli-Q water in a glass beaker with a volume of 600 mL. Soil samples were pre-concentrated and purified using OASIS HLB cartridges (200 mg; 6 mL; particle diameter 25–35 μm; WATERS, USA) using a Baker vacuum system (J.T. Baker, Deventer, The Netherlands). The SPE protocol was inspired by Gibs et al. (2013) and Sun et al. (2017), which used OASIS HLB cartridges for extraction of antibiotics as well. Briefly, the SPE column was conditioned by 6 mL of methanol (MeOH), followed by 6 mL of Milli-Q water. Consequently, diluted soil extract was loaded on the SPE column. Subsequently, the washing step was performed with 15 mL of Milli-Q water, followed by cartridge air-drying for 2 min. Elution was performed by 9 mL of 0.1% formic acid (FA) in MeOH, followed by evaporation under the nitrogen stream almost to dryness (thermostat heated to 40 °C). Finally, 990 μL of 0.1% FA in H_2_O:ACN (95:5) and 10 μL of a mixture of internal standards (mixture concentration 10 μg∙mL^−1^) were added. Before LC–MS/MS analysis, the samples were filtered through 0.22-μm nylon syringe filters (diameter 13 mm). Subsequently, the FQ concentrations in the soil samples were calculated according to Eq. 6.

6 $$\mathrm{FQs in soil}=\frac{\mathrm{Measured analyte concentration }\bullet {V}_{{\text{reconstitution}}}}{\mathrm{Sample weight}}$$

where *V*_reconstituion_ is the volume of soil extract after nitrogen drying; addition of IS and solvent reconstitution (1 mL).

Method validation results for poultry litter can be found in Table 2 and those for soil in Table 3. For the quantification of FQs in poultry litter and soil samples, an external calibration method with isotopically labelled internal standards was used to compensate for instrument drift in sensitivity and for matrix effects (the IS does not compensate RR, as it was added before sample filtration). The calibration range was 0.1 to 500 ng∙mL^−1^ for both FQs. The coefficients of determination (*R*^2^) for ENR and CIP were > 0.995. The instrumental LoD and LoQ were calculated according to Eqs. 2–3 (method based on the standard deviation of the response and the slope), and these instrumental limits were subsequently recalculated to the method limits. The method recovery rates (RR%) for poultry litter were determined by spiking blank poultry litter at five concentration levels (1.0; 2.5; 5.0; 25; 50 mg∙kg^–1^) and calculated using Eq. 7. The method RR % for soil were determined by spiking blank soil at three concentration levels (50; 100; and 500 μg∙kg^–1^) and calculated using Eq. 8. The matrix effects (% ME) were determined using Eq. 9, as the ratio of the analyte signal post-extraction spiked blank matrix and analyte signal in the solvent. The trueness is expressed as recovery rate (RR %); intra-day precision and inter-day precision of the method are calculated in terms of RSD% of the RR (Eq. 10); however, accuracy and precision were also checked by analysis of the QC samples within each LC–MS run. The selectivity/specificity of the method was assessed by analysing poultry litter and soil blank samples. Confirmation of identity was achieved by monitoring the ion ratio of multiple MS/MS transitions in the MRM mode during LC–MS/MS analysis.

7 $$\mathrm{Recovery rate for poultry litter }(\mathrm{\% RR})=\frac{\mathrm{Measured analyte concentration }\bullet {V}_{\mathrm{total of extract }}\bullet {F}_{{\text{dilution}}}}{\mathrm{Spiked analyte concentration}}\bullet 100$$

where *V*_total of extract_ is the total volume used for extraction (30 mL) and *F*_dilution_ is the factor of dillution used to dilute the sample before IS spiking, sample filtration, and analysis (0 or 10).

**Table 2:** Method validation for poultry litter samples

Analyte name	LoD (μg∙kg^−1^)	LoQ (μg∙kg^−1^)	RR (%) 1.0 mg∙kg^−1^	RR (%) 2.5 mg∙kg^−1^	RR (%) 5.0 mg∙kg^−1^	RR (%) 25 mg∙kg^−1^	RR (%) 50 mg∙kg^−1^	RSD (%)	ME (%)
ENR	105	321	106 ± 8	121 ± 3	103 ± 56	123 ± 4	115 ± 2	4	142 ± 11
CIP	189	573	111 ± 9	130 ± 4	110 ± 9	120 ± 7	112.7 ± 0.4	5	142 ± 10

**Table 3:** Method validation for soil samples

Analyte name	LoD (μg∙kg^−1^)	LoQ (μg∙kg^−1^)	RR (%) 50 μg∙kg^−1^	RR (%) 100 μg∙kg^−1^	RR (%) 500 μg∙kg^−^	RSD (%)	ME (%)
ENR	0.35	1.07	127 ± 11	109 ± 6	67 ± 1	6	121 ± 26
CIP	0.63	1.91	79 ± 9	68 ± 5	80 ± 2	7	109 ± 13

8 $$\mathrm{Recovery rate for soil }\left(\mathrm{\% RR}\right)=\frac{\mathrm{Measured analyte concentration}}{\mathrm{Spiked analyte concentration}}\bullet 100$$

9 $$\mathrm{Matrix effect }(\mathrm{\% ME})=\frac{{\mathrm{Analyte signal}}_{{\text{post}}-\mathrm{extraction spiked matrix}}}{{\mathrm{Analyte signal}}_{{\text{solvent}}}}\bullet 100$$

10 $$\mathrm{RSD \%}=\frac{\mathrm{Standard deviation of RR\%}}{\mathrm{Mean RR\%}}\bullet 100$$

### LC–MS/MS method for quantification of FQs

Instrumental determination of FQs was performed using ultra-performance liquid chromatography (UHPLC Agilent 1290 Infinity LC) in tandem with triple quadruple (Bruker EVOQ LC-TQ) with atmospheric pressure electrospray ionisation (ESI). As the source of nitrogen and air, an external generator of gases was used (Peak Scientific–Genius 3045).

Chromatographic separation was achieved with Luna® Omega Polar C18 Phenomenex (100 × 2.1 mm; 1.6 µm) column. The optimum column temperature was adjusted to 35 °C and the flow rate was set to 0.5 mL∙min^−1^. As the mobile phases: (A) 0.1% FA in H_2_O and (B) ACN were used with the following gradient programme of A eluent (%): *t* (0 min) = 90, *t* (0.5 min) = 90, *t* (13.0 min) = 65, *t* (13.1 min) = 10, and *t* (15.5 min) = 90. The stop time of the LC method was set to 16 min, and the re-equilibration time was set to 2 min. The injection volume applied in all analyses was 7 µL. To minimize carry-over, the flush port function was used for 30 s and the composition of the solvent mixture was FA:H_2_O:ACN at a ratio of 1:9:90.

MS conditions were as follows: electrospray ionisation in positive mode: spray voltage: 4500 V; cone temperature: 350 °C; cone gas flow: 15 a.u.; heated probe temperature: 500 °C; probe gas flow: 25 a.u., nebulizer gas flow: 45 a.u.; and exhaust gas: ON. For quantitative and qualitative analysis of FQs, MRM mode was used with the following MRM transitions: ciprofloxacin (retention time (Rt) 3.41 min) 332.4 > 287.8 (15 eV) and 332.4 > 245.0 (15 eV); ciprofloxacin-d8 (Rt 3.41 min) 340.4 > 322.4 (15 eV) and 340.4 > 296.1 (15 eV); enrofloxacin (Rt 3.81 min) 360.4 > 244.9 (20 eV) and 360.4 > 316.1 (15 eV); and enrofloxacin-d5 (Rt 3.81 min) 365.4 > 245.2 (25 eV) and 365.4 > 320.9 (10 eV). As the collision gas, argon was used at a pressure of 1.5 mTorr.

### PCR analysis of ARGs

The detection of selected genes was performed in samples of poultry litter and soil. For the analysis, 25 g of each sample was homogenized in 225 mL Buffered Peptone Water (Oxoid, UK) and incubated at 37 °C overnight under aerobic conditions. To reduce the possibility of detecting DNA from dead bacterial cells, DNA was extracted from 1 mL enriched suspension using QIAamp Fast DNA Stool Mini Kit (Qiagen, Germany) following the instructions.

The presence of genes encoding Qnr proteins *qnrA*, *qnrB*, and *qnrS* (Cattoir et al. 2007), aminoglycoside acetyltransferase *aac(6´)Ib–cr* (Park et al. 2006), and efflux pump *qepA* (Yamane et al. 2008) was screened by PCR in separate reactions. The reaction system was adopted from previous studies (Cattoir et al. 2007; Park et al. 2006; Yamane et al. 2008) with modifications using a QIAGEN Multiplex PCR Kit (Qiagene, Germany). To prevent the effect of possible PCR inhibitors, the extracted DNA was diluted 1:10 with sterile PCR water.

## Results and discussion

### Method development for extraction of FQs from soil

In the case of method development, 1 g of soil was spiked with FQs (500 ng in 500 μL of MeOH) and left overnight to evaporate. The following day, the extraction was carried out according to the optimized extraction method described in the “Extraction of fluoroquinolones from poultry litter and soil samples” section, with varying parameters of the composition of the extraction medium (the “Influence of the composition of different extraction media on recovery rate” section) or temperature of the ultrasound bath (the “Influence of extraction temperature in ultrasound bath on recovery rate” section).

#### Influence of the composition of different extraction media on recovery rate

**Fig. 1:**
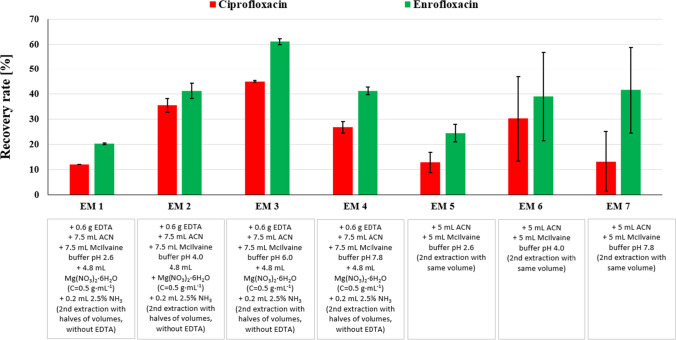
Influence of the composition of different extraction media on the RR (%); EM, extraction medium; RR, recovery rate

Used extraction media (EM) and extraction parameters were inspired by studies Albero et al. (2018); Huang et al. (2013); Silva et al. (2020); Sun et al. (2017); Yu et al. (2012), where a mixture of MgNO_3_ or citrate–phosphate buffer (McIlvane buffer) of different pH values with organic solvent (acetonitrile or methanol) was used for extraction of FQs or other PhACs. Due to the zwitterionic character of FQs, different extraction media with varying pH values were used with the following recovery rates (Fig. 1).

Polarity, solubility, Kow values, stability under acidic and basic conditions, and especially pKa values (Table S1) can influence the RR of extracted substances (Huang et al. 2013). Therefore, pH of the aqueous buffer can greatly influence the extraction efficiency, because of different charges of the analytes and soil substances. In addition, the composition of EM can influence the type of extraction mechanism, e.g., in the case of the MgNO_3_ extraction mechanism is metal complexation (Turiel et al. 2006).

ANOVA and Tukey’s HSD tests (Table S4 and Table S5) were carried out to investigate, whether the pH and extraction mechanism have a significant effect on the extraction efficiency. During the evaluation of the pH effect (MgNO_3_ solution–extraction via metal complexation), significant differences (*p* < 0.05) were observed when comparing pH 2.6 (EM1) vs pH 4.0 (EM2) and pH 2.6 (EM1) vs pH 6.0 (M3) for both ENR and CIP. Meanwhile, pH 2.6 (EM1) vs pH 8.0 (EM4) did not show significant difference (*p* > 0.05), which can be explained by the inverted U dependency of RR on pH. In addition, no significant differences (*p* > 0.05) were observed when carrying out extraction with McIlvaine buffer (M5-M7), which can be explained by high standard deviations of RR and overall unsuitability of this extraction mechanism for FQs.

As can be seen in Fig. 1, the lowest RR (%) was achieved with McIlvaine buffer of pH 2.6 with both EM, which can be explained by FQs having a positive charge at acidic pH (due to their pKa) (Uslu et al. 2007), whilst soil substances have a primarily negative charge. The rise in pH of the aqueous buffer is followed by an increase in RR in both EMs for both FQs, as these substances become more neutral (are at their zwitterion form) or negatively charged due to changes in pH. If we compare different extraction mechanisms (EM3 and EM6), the extraction of FQs via metal complexation is more suitable, which is in agreement with Turiel et al. (2006). The most efficient in extraction of FQs was EM3 (pH 6.0), where RR (61.1 ± 1.6)% for ENR and RR (45.1 ± 2.9)% for CIP were achieved. However, this contradicts the findings of studies Carballo et al. (2007) and Qassim et al. (2020), where the most efficient EM had an acidic pH. Meanwhile, in Silva et al. (2020), higher recovery rates of ENR were achieved with an aqueous buffer with basic pH. Contradicting results of pH influence on recovery rates of PhACs across scientific studies are probably caused by different soil properties, which are not usually reported within scientific study (properties of our soil can be found in Table S2 and Table S3). Furthermore, Na_2_EDTA is commonly added to EM to increase RR; however, according to Wei et al. (2016), excess amounts of EDTA can chelate not only metals but also organic compounds of interest.

#### Influence of extraction temperature in ultrasound bath on recovery rate

**Fig. 2:**
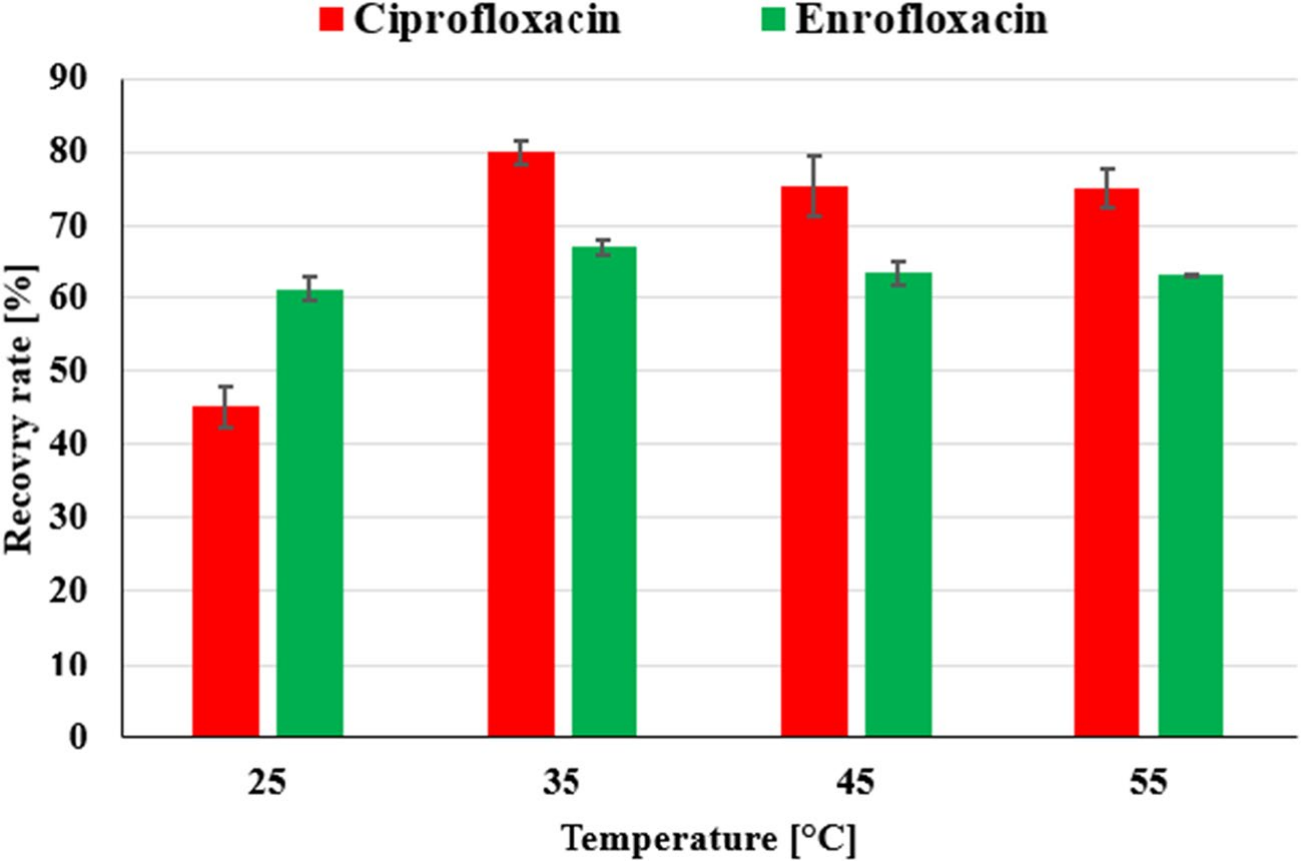
Influence of the extraction temperature in ultrasound bath on the RR (%)

The influence of extraction temperature in the ultrasound bath was carried out using EM3. A study (Ferrero et al. 2015) stated that temperature is the key extraction condition to optimize due to its impact on RR (%). Therefore. extraction at temperatures 25, 35, 45, and 55 °C was carried out, as can be seen in Fig. 2. The results showed that the extraction temperature has a great impact on the extraction equilibrium due to the enthalpy and solubility of PhACs.

The lowest RR (%) was achieved at 25 °C and the highest RR (%) at 35 °C: (80.0 ± 1.6)% for CIP and (67.1 ± 1.0)% for ENR. The increase in temperature to 45 and 55 °C has a slightly negative influence on RR (%), which can be caused by degradation or a negative impact on extraction equilibrium. In addition, ANOVA and Tukey’s HSD tests were carried out to investigate, whether temperature has a statistically significant effect on RRs (Table S6 and Table S7). For ENR, only a statistical difference between temperatures 25 and 35 °C was observed (*p* < 0.05). Meanwhile, analysis of RRs of CIP suggested differences of temperature of 25 °C with all others (*p* < 0.05). Even though the comparison of other temperatures did not show significant differences (*p* > 0.05), the mean differences were the highest for temperature 35 °C for both compounds. These results are in agreement with those of Ferrero et al. (2015), as temperature has a great influence on RR (%) and contradicts the findings of Turiel et al. (2006), who claimed that temperature has only a negligible effect on RR (%).

### FQs in medicated drinking water and poultry litter

Samples of drinking water contained (41.0 ± 0.3) mg∙L^−1^ of ENR at the beginning of the pipeline (*N* = 3) and (32.8 ± 0.3) mg∙L^−1^ of ENR at the end of the pipeline (*N* = 3); no statistically significant differences were suggested through a t-test as *p* > 0.05. A lower concentration of ENR at the end of the pipeline can be caused by sorption in PE plumbing, as Mompelat et al. (2013) and Petrovic (2014) suggest that PhACs can adsorb to different materials. CIP was under LoD in all samples of drinking water, meaning that ENR was not degraded to CIP before administration to broilers. The daily dose of ENR for a broiler is supposed to be 10 mg∙kg^−1^ body weight/day, when we suppose that the average broiler drinks 200 mL of water/day (Temmerman et al. 2021); then the concentration of ENR in medicated drinking water should be around 50 mg∙L^−1^ for this dose, which is in agreement with our results.

**Fig. 3:**
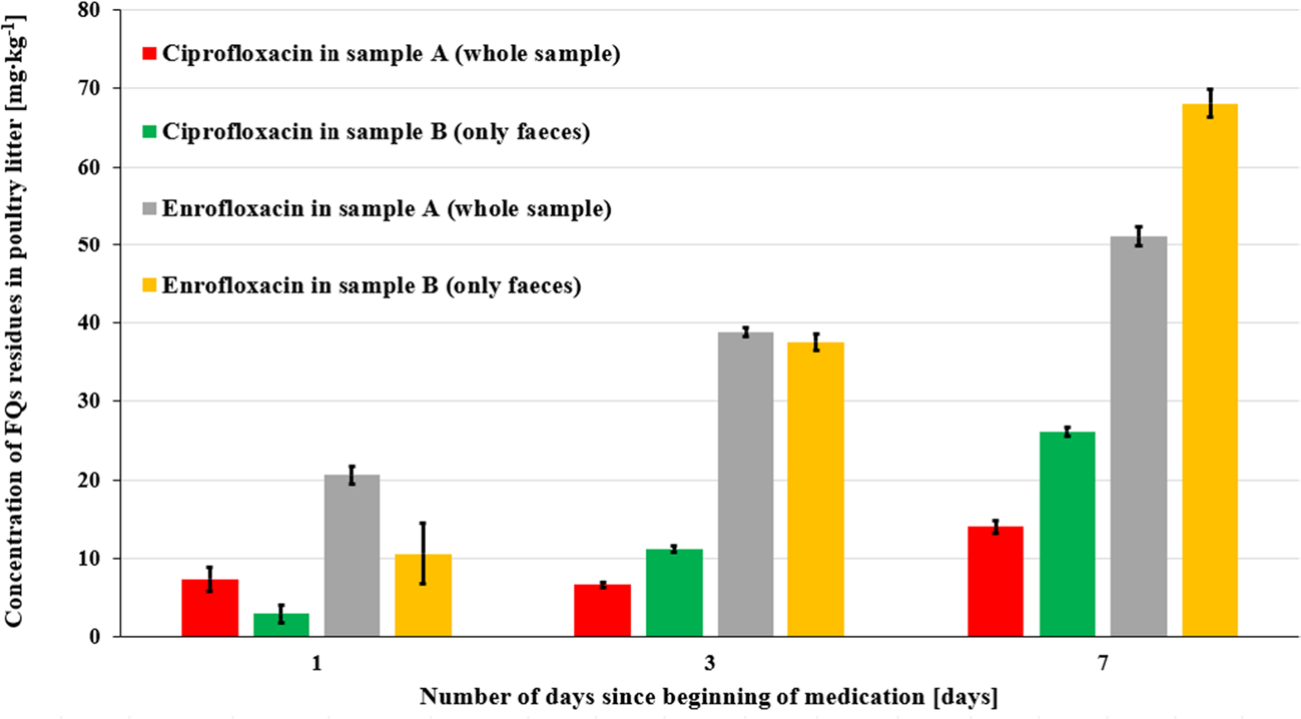
Concentrations of FQs in poultry litter samples and faeces

Poultry litter samples (*N* = 3) were separated into subsamples A (only faeces) and B (whole poultry sample); these samples were analysed by the extraction method without SPE as described in the “Extraction of fluoroquinolones from poultry litter and soil samples” section. Unlike in the case of medicated drinking water, the metabolite CIP was present in all samples (except the BLANK sample). The presence of CIP as a metabolite is in agreement with Gutiérrez et al. (2015), where 31 different metabolites of ENR in broiler chickens’ tissues were identified. The results (Fig. 3) prove that a high amount of ENR is excreted as a parent drug or as a still active metabolite (CIP). The presence of CIP in poultry litter can also be caused by photodegradation or microorganisms. Due to the four-day medication of broilers with ROXACIN, the concentrations of FQs in poultry litter were gradually rising, as the amount of faeces was increasing with the treatment/medication time of broilers (e.g., in the case of whole poultry litter samples from day 1; 3; and 7; the ENR concentrations were 20.6, 38.8, and 51.1 mg∙kg^−1^ respectively). To the best of our knowledge, no study has investigated the concentrations of FQs in poultry litter at different treatment time, although Reyes-Herrera et al. (2011) have observed increasing concentrations of ENR in chicken muscle (up to 1.6 mg∙kg^−1^) and blood (up to 0.8 mg∙kg^−1^) over a dosing period of 7 days. In addition, two-way ANOVA was performed to assess if there was a significant difference between antibiotics (ENR and CIP), sampling times (1; 3; and 7 days), and sample types (whole poultry litter and only faeces). Significant differences between antibiotics (*p* < 0.05) and between sampling times (*p* < 0.05) were observed. Meanwhile, no significant difference between sample types was found (*p* > 0.05). Furthermore, when considering interactions, only the combination of antibiotics:sampling time suggested significant interaction (*p* < 0.05). Subsequently, the Pearson correlation coefficient proved positive correlations of CIP to ENR in faeces (*r* = 0.99) and in whole poultry litter sample (*r* = 0.75).

The concentrations of FQs in poultry litter are lower than those in study by Zhao et al. (2010) where ENR concentrations ranged from 0.33 to 1420.76 mg∙kg^−1^ and CIP concentrations ranged from 0.68 to 45.59 mg∙kg^−1^ of poultry litter. Our results (in concentration) are much higher than those in the scientific study by Hou et al. (2014), where ENR concentrations ranged from (38.8–278.7) μg∙kg^−1^; CIP concentrations ranged from (30.7–91.8) μg∙kg^−1^ and then in the study Zhang et al. (2014), where ENR concentrations ranged from (10.6–8575) μg∙kg^−1^ and CIP concentrations ranged from (19.1–4905) μg∙kg^−1^. Concentrations of FQs in poultry litter are widely different across studies. This can be caused by several phenomena, such as different medication concentrations in water, number of days since the beginning of medication, maturation age of poultry litter, sampling method, analytical methods, etc. Furthermore, contamination by FQ residues and resistance genes can be further reduced by manure processing before its application to terrestrial environments (Du and Liu 2011; Xu et al. 2020). In the study by Slana et al. (2017), approximately 66% of ENR and 92% of CIP were removed from poultry litter after 60 days. Overall, the efficiency of VA removal is usually in the range of 40–100% depending on the chosen method of manure processing (composting, vermicomposting, and aerobic or anaerobic fermentation), processing time (2 weeks to several months), processing conditions (aerobic/anaerobic, temperature, humidity, etc.), and properties of the veterinary drug (Mohring et al. 2009; Motoyama et al. 2011; Slana et al. 2017).

### FQs in soil samples

**Fig. 4:**
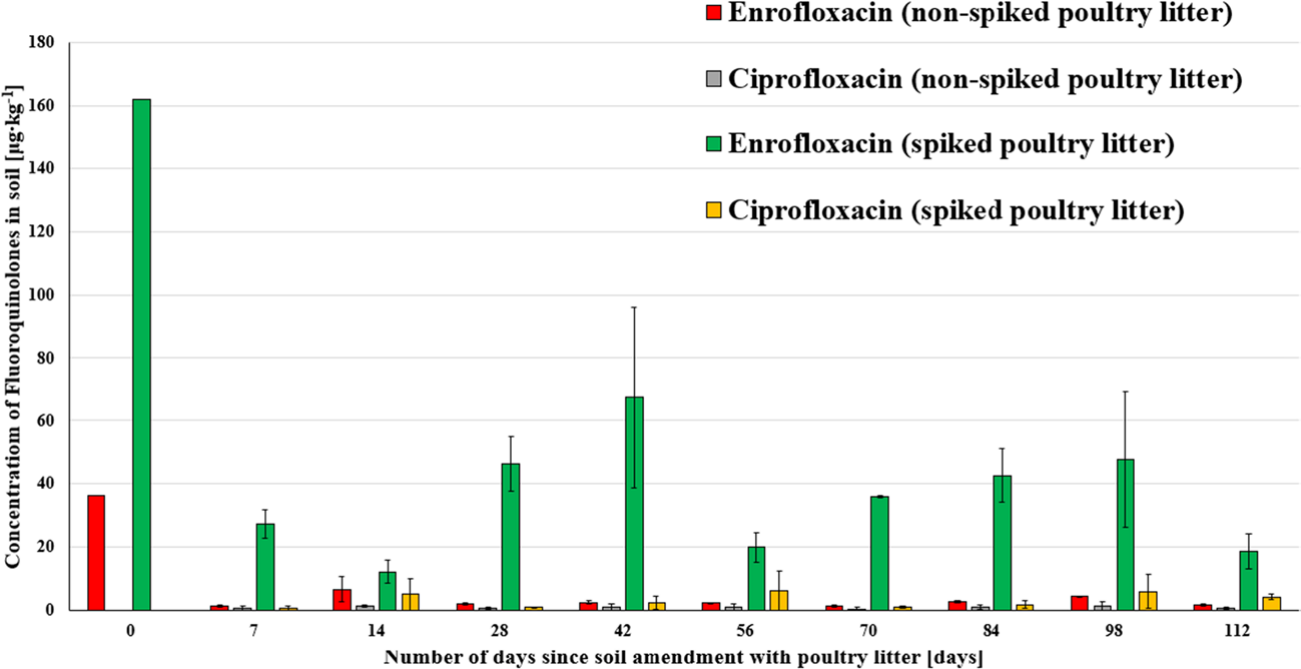
Soil concentrations of fluoroquinolones after amendment with poultry litter at the experimental field during 112 days

21 soil samples were analysed by the extraction method described in the “Extraction of fluoroquinolones from poultry litter and soil samples” section. FQs were detected in all soil samples from day 0 to day 112 (Fig. 4; *N* = 2). Soil where spiked poultry litter was applied contains approximately about 100 μg∙kg^−1^ of ENR more than soil amended with non-spiked poultry litter (day 0 at Fig. 4), as was experimentally planned to investigate the persistence of FQs at two different concentrations level. ENR and CIP were present in all soil samples on which poultry litter was applied, with the exception of CIP not being present in the soil at day 0. The absence of CIP in the first soil samples can be explained by its lower concentration in poultry litter (Fig. 3).

The initial concentrations of ENR (36.3 μg∙kg^−1^) in soil (after incorporation of naturally contaminated poultry litter) are lower than in the study by Arun et al. (2020), where the CIP concentration was measured to be (33 ± 26) mg∙kg^−1^; in the study by Carballo et al. (2007), the CIP concentration in soil reached up to 0.37 mg∙kg^−1^; and in the study by Wei et al. (2016), the ENR concentration was high as 1420 mg∙kg^−1^. Similar FQ concentrations were found in the study by Uslu et al. (2007), where the ENR concentration ranged from 13 to 204 μg∙kg^−1^ and in the study by Yang et al. (2016), where the summarized accumulation of fluoroquinolone antibiotics (norfloxacin, ofloxacin, ciprofloxacin, and enrofloxacin) was 237–336 μg∙kg^−1^. Concentrations of FQs in terrestrial environments are widely different across studies. This can be caused by several factors, such as sampling location (dumpsite soil, agricultural field, etc.), sampling time since animal manure incorporation, animal manure processing method and its efficiency, amount of incorporated animal manure, sampling method, analytical method, etc. (Arun et al. 2020; Carballo et al. 2007; Uslu et al. 2007; Yang et al. 2016). Furthermore, the concentration of VAs in soils can also be elevated by repeated application of animal manure (Kim et al. 2010).

Concentrations of ENR were gradually decreased with time (at both concentration levels), although ENR was still persistent in the soil environment, even after more than 90 days. Nevertheless, we can say that the concentrations of CIP were constant from days 7 to 112, which means that CIP is persistent in the soil environment as well. However, the concentration of CIP in soil ranged only between 1 and 7 μg∙kg^−1^, which are concentrations between LoD and LoQ or above LoQ. Decreasing concentrations of FQs over time can be caused by naturally occurring degradation, by microbial degradation, or by their uptake by Scorpion Weed (*Phacelia tanacetifolia*) grown in the experimental field during our experiment. One-way repeated measures ANOVA showed significant differences between the levels of ENR (*p* < 0.05) and CIP (*p* < 0.05) for soil with spiked and non-spiked poultry litter over time. The persistence of FQs is in agreement with Albero et al. (2018), where both ENR and CIP DT_50_ were estimated to be > 90 days; Boxall et al. (2006) estimated ENR DT_50_ > 152 days; and Sanford et al. (2009) estimated the half-lives of FQs to be 100 days. Degradation time of VAs is dependent on drug properties (pKa, Kd), physicochemical properties of soil (e.g. pH, content of organic matter, concentrations of divalent cations such as Ca^2+^ and Mg^2+^), presence of microorganisms, and experimental conditions (temperature, the timing of manure application, weather, etc.). In addition, VAs can be uptaken by soil organisms or crops grown on agricultural fields (Kim et al. 2010; Sanford et al. 2009).

Data from our meteorological station (Fig. S1) shows that there was more rainfall during August (74th to 103rd day of the experiment) than in other months. Similar to Niemi et al. (2020), linear modelling (Fig. S2) was performed to assess whether a significant correlation existed between total rainfall and FQ concentrations in soil. Analysis showed no significant correlation (*p* > 0.05), which is in agreement with Huang et al. (2013) and Yu et al. (2012), as FQs are strongly sorbed to soil (FQs have Kd between 260–6310 L∙kg^−1^) and not leached out (Sanford et al. 2009; Song and Guo 2014). The high persistence of FQs in the soil environment presents a suitable environment for the uprise of AMR. The presence of ARGs for FQs in our soil sample was also investigated, and the results can be found in the “Preliminary results on the occurrence of ARGs in poultry litter and soil environment” section. The presence of VA in the soil environment not only increases drug resistance but also affects the metabolism of soil microbial communities. Unfortunately, any change in the microbial communities may influence the regular functioning of the soil ecosystem (Butler et al. 2012; Fatta-Kassinos et al. 2010; Rehman et al. 2015).

The effects of PhACs on the aquatic environment have been closely investigated, although there are very few scientific articles regarding the ecotoxicology of PhACs in the soil environment (Gworek et al. 2021). ENR belongs amongst the most toxic antibiotics for aquatic species (Gworek et al. 2019). The International Cooperation on Harmonisation of Technical Requirements for Registration of Veterinary Medicinal Products (VMPs) proposed that the total concentration of VMPs in the soil environment should not exceed 100 μg∙kg^−1^, as at this level, ecotoxicity tests with VMPs showed no effects on earthworms, microbes, or plants (Kim et al. 2010; Rehman et al. 2015). This limit was not exceeded with the amendment of naturally contaminated (non-spiked) poultry litter; however, as mentioned, many studies have found higher concentrations, which is an alarming state, as animal manure, biosolid, or wastewater is commonly applied to agriculture fields.

Furthermore, the potential environmental risk in fertilized soil by non-spiked poultry litter was evaluated. PNEC_soil_ (predicted no-effect concentration) was calculated using Eq. 11 (Sun et al. 2017), since PNEC_soil_ values are not available due to the lack of ecotoxicological data for terrestrial environments.

11 $${{\text{PNEC}}}_{{\text{soil}}}={{\text{PNEC}}}_{{\text{water}}}\cdot{\text{Kd}}$$

where PNEC_soil_ is the predicted no-effect concentration in soil, PNEC_water_ is the predicted no-effect concentration, and Kd is the distribution coefficient for a given compound in the soil environment.

The following values were used to calculate PNEC_soil_: PNEC_water, ENR_ = 1.9 μg∙L^−1^ (Amr Alliance science-based PNEC targets for risk assessments (2023)); PNEC_water, CIP_ = 0.45 μg∙L^−1^ (Amr Alliance science-based PNEC targets for risk assessments (2023)); and Kd values (Kd_ENR_ = 429.8 L∙kg^−1^ and Kd_CIP_ = 569.4 L∙kg^−1^) (Sun et al. 2017). In addition, study Amr Alliance science-based PNEC targets for risk assessments (2023) has published PNEC_WATER,MIC_ (PNEC–Minimum Inhibitory Concentration) values which can provide insight into the potential risk towards the uprise of AMR (PNEC_water, MIC-ENR_ = 0.06 μg∙L^−1^; and PNEC_water, MIC-CIP_ = 0.06 μg∙L^−1^). The transferability of PNEC values from aquatic conditions to terrestrial conditions depends on various factors, such as different exposure routes to organism, different behaviour of VAs in water and soil (especially wide range of Kd values due to varying soil properties), different organism sensitivity to contaminants, and various environmental conditions (temperature, pH, organic matter, etc.). Although, this estimation should be sufficient for conservative ecological risk assessments, as it is commonly used due to the lack of data for soil environment (Carlsson et al. 2006; Deschamps et al. 2017; Parente et al. 2019a, b; Sun et al. 2017). The following PNEC values were obtained for terrestrial values: PNEC_SOIL,ENR_ = 816.6 μg∙kg^−1^; PNEC_SOIL,MIC-ENR_ = 25.8 μg∙kg^−1^; PNEC_SOIL,CIP_ = 256.2 μg∙kg^−1^; and PNEC_SOIL,MIC-CIP_ = 34.2 μg∙kg^−1^. Subsequently, RQ values were calculated as the ratio of the measured environmental concentration (MEC) and a predicted no effect concentration (PNEC); all calculated RQs can be found in Table S8. The criteria for the interpretation of RQs were those commonly used: low risk when RQ < 0.1, medium risk when 0.1 < RQ < 1, and high risk for RQ > 1 (Deschamps et al. 2017; Sun et al. 2017). The obtained RQ_soil_ values show that in the case of ENR and CIP, the potential environmental risk for organisms was immediately low, as all RQ_soil_ values were < 0.1. However, the potential risk for uprise of AMR was present for over 115 days, as RQ_soil,MIC-ENR, day 0_ = 1.4, with a rapid decrease of this risk to RQ_soil,MIC-ENR, day14_ = 0.3, and subsequently to RQ_soil,MIC-ENR,day 28_ = 0.1 with constant value until the end of field experiment (day 115). Meanwhile, the potential risk for uprise of AMR due to CIP was not present, as RQ_soil,MIC-CIP_ = 0 for the entire duration of field experiment. Even these low concentrations of ENR in the soil environment can alter soil microbial community and pose an environment for AMR increase, which is in agreement with our preliminary results on the occurrence of ARGs.

### Preliminary results on the occurrence of ARGs in poultry litter and soil environment

Four samples were selected for PMQR gene detection. Their presence was screened in poultry litter sampled in the breeding hall, in the soil from the experimental field before the application of poultry litter, and from both parts of the experimental field one week after poultry litter application and spiking. The results shown in Table 4 reveal that three out of five tested PMQR genes were detected in poultry litter contrary to soil before the experiment, which was negative for all screened genes. In the soil after incorporation of poultry litter (both natural and spiked poultry litter) only *aac(6´)Ib–cr* gene was positive.

**Table 4:** The presence of plasmid-mediated quinolone resistance genes (PMQR) in examined samples

Sample description	PMQR genes				
	*qnrA*	*qnrB*	*qnrS*	*aac(6´)Ib–cr*	*qepA*
Poultry litter	−	+	+	+	−
Soil before application of poultry litter	−	−	−	−	−
Soil 1 week after application of poultry litter	−	−	−	+	−
Soil 1 week after spiking FQs	−	−	−	+	−

Genes encoding the Qnr proteins *qnrA*, *qnrB*, and *qnrS* were reported to be the most prevalent and were carried not only by the bacteria family *Enterobacteriaceae* but also *Aeromonadaceae* or *Vibrionaceae* (Marti and Balcázar 2013). They were found in wastewater from hospitals or veterinary clinics, animal manure, and soil (Marti et al. 2012; Mu et al. 2014; Rusu et al. 2014). The persistence of resistance genes in soil depends on factors such as native reservoirs, manure microbiota, and the presence of mobile gene elements (Xu et al. 2015). A study by Pourcher et al. (2014) reported that ciprofloxacin-resistant *Escherichia coli* survived in soil fertilized with chicken manure for at least three months. In our study, these genes were found only in poultry litter compared with genes encoding different types of resistance mechanisms—aminoglycoside acetyltransferase *aac(6´)Ib–cr*. The gene has been shown to influence the phenotypic resistance of only specific quinolones, ciprofloxacin, and norfloxacin, with respect to their chemical structure (Strahilevitz et al. 2009). Antimicrobial resistance genes are to be naturally present in soil microbiota, but their occurrence is also related to soil contamination by antibiotics. A direct link between antibiotic concentration in soil and a specific gene is difficult to observe given the complexity of natural ecosystems (Caracciolo et al. 2022). In fact, studies have shown that ARGs can spread or persist even when the antibiotic is fully or partially degraded (Parente et al. 2019a, b; Wang et al. 2021). As shown in the study by Caracciolo et al. (2022), gene abundance varies across microbial populations and differs in the way they spread. Genes carried on small, unbound plasmids with a wide range of recipients can replicate in many bacterial hosts.

## Conclusion

This study has provided valuable insights into the entry and fate of fluoroquinolones, specifically enrofloxacin and ciprofloxacin, in the terrestrial environment through an in-depth analysis of medicated drinking water, poultry litter, and soil samples over a span of 112 days. The comprehensive investigation covered the entire lifecycle of these antimicrobials, shedding light on their persistence, degradation patterns, and potential environmental risks. Furthermore, ARG analysis was conducted on both poultry litter and soil samples.

In this scientific study, a robust and viable method for analysing selected fluoroquinolones in medicated drinking water, poultry litter, and soil samples is introduced, employing LC–ESI–MS/MS analysis. The recoveries calculated from spiked samples demonstrated a high level of accuracy, ranging from 103.1 to 130.3% in poultry litter with RSD 4% for ENR and 5% for CIP. Meanwhile, recovery rates for soil samples ranged from 67.1 to 126.6% in soil, with RSD 6% for ENR and 7% for CIP.

Even though this study presented one of the worst-case scenarios, as poultry litter was treated insufficiently before its incorporation into soil, our data shows that FQs undergo degradation over time. Although FQs are persistent micropollutants (> 100 days), ARGs encoding resistance to FQs were already present 7 days after the incorporation of poultry litter into the soil. These results are in agreement with risk assessment toward AMR uprise in the soil environment. The implications extend to global environmental and public health, given the already significant number of deaths attributed to AMR and the potential escalation of these numbers.

The data obtained in this study prove the necessity of developing treatment methods for animal manure and policy before its incorporation into agricultural fields and adequate regulations of pharmaceuticals in animal manure and terrestrial environment. Otherwise, the dissemination of pharmaceuticals and ARGs into the environment can result in environmental and health risks, as these substances can also enter the food chain after their uptake by agricultural crops.

### Supplementary Information

Below is the link to the electronic supplementary material.

Supplementary file1 (DOCX 351 KB)

## Data Availability

The data that support the findings of this study are available from the corresponding author, [Jan Fučík], upon reasonable request.
