# Supplementary material for: Fate of fluoroquinolones in field soil environment after incorporation of poultry litter from a farm with enrofloxacin administration via drinking water
Source: Environ Sci Pollut Res Int. 2024 Feb 17;31(13):20017–32. doi: 10.1007/s11356-024-32492-x (PMC10927849; doi:10.1007/s11356-024-32492-x)
Supplement: Supplementary file 1 — Supplementary file1 (DOCX 351 KB) [file 11356_2024_32492_MOESM1_ESM.docx]

**Supporting information for:**

**Fate of fluoroquinolones in field soil environment after incorporation of poultry litter from a farm with enrofloxacin administration via drinking water**

Jan Fučík^1^,^*^, Anna Amrichová^1^, Kristýna Brabcová^1, 4^, Renata Karpíšková^2^, Ivana Koláčková^2^, Lucie Pokludová^3^, Šárka Poláková^4^, Ludmila Mravcová^1^

*corresponding author:[xcfucikj@vutbr.cz](mailto:xcfucikj@vutbr.cz)

1 Institute of Environmental Chemistry, Faculty of Chemistry, Brno University of Technology, Purkyňova 118, 612 00 Brno, Czech Republic

2 Department of Public Health, Faculty of Medicine, Masaryk University, Kamenice 5, 625 00 Brno, Czech Republic

3 Institute for State Control of Veterinary Biologicals and Medicines (ISCVBM), Hudcova 56 A, Brno, Czech Republic

4 Central Institute for Supervising and Testing in Agriculture (UKZUZ), Hroznová 63/2, Brno, 603 00, Czech Republic

**Supporting Information includes:**

Section 1: Chemical properties of selected compounds

Section 2: Physico-chemical properties of soil and meteorological data from experimental field

Section 3: Statistical Analysis of the Influence of Extraction Parameters on Recovery Rate

Section 4: Correlation between Cumulative Rainfall and Fluoroquinolone concentrations in the soil

Section 5: Calculated RQ values for ENR and CIP

In total: 7 pages, 8 tables, 1 texts, 2 figures, and references

**Section 1:** Chemical properties of selected compounds

**Table S1** Chemical properties of Enrofloxacin and Ciprofloxacin (Boxall et al. 2006; Sanford et al. 2009)

| Name of the compound | CAS | Structure | Formula | Solubility in water [g∙L^-1^] | Molecular weight [-] | pKa_1_ [-] | pKa_2_ [-] | logKow [-] | Kd [L∙kg^-1^] |
| --- | --- | --- | --- | --- | --- | --- | --- | --- | --- |
| Enrofloxacin | [93106-60-6](https://commonchemistry.cas.org/detail?cas_rn=93106-60-6) | 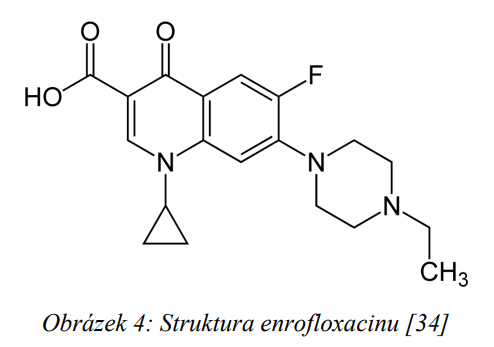 | C_19_H_22_FN_3_O_3_ | 130 | 359.4 | 6.27 | 8.3 | 1.1 | 260 – 6,310 |
| Ciprofloxacin | [85721-33-1](https://commonchemistry.cas.org/detail?cas_rn=85721-33-1) | 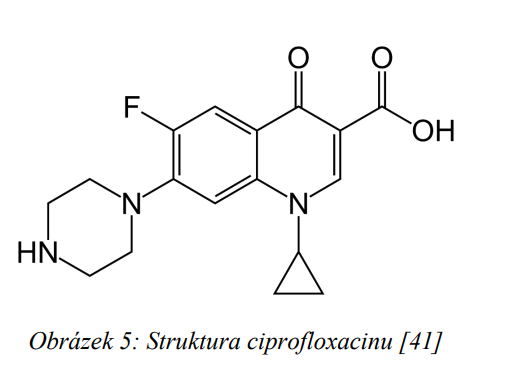 | C_17_H_18_FN_3_O_3_ | 30 | 331.3 | 5.9 | 8.89 | 0.4 | 430 |

**Section 2:** Physico-chemical properties of soil and meteorological data from experimental field

The soil type in our experimental field is fluvial. Based on the analysis of the sample (see Table S2), it is medium-grained soil with neutral pH and high to very high levels of available nutrients. The total carbon and nitrogen contents were also determined in the sample. These correspond to the range of arable land. The C:N ratio is optimal. The content of the elements at risk is higher than the median value for arable soils calculated from data on long-term monitoring plots (see Table S3), but higher element and substance contents are typical for fluvious. Furthermore, meteorological conditions were monitored during the experiment (see Figure S1).

**Table S2** Agrochemical properties of soil

|  | pH/CaCl_2_ [-] | P [mg∙kg^-1^] | K [mg∙kg^-1^] | Mg [mg∙kg^-1^] | Ca [mg∙kg^-1^] | N_tot_ [%] | TOC [%] |
| --- | --- | --- | --- | --- | --- | --- | --- |
| 2021 | 7.1 | 161 | 315 | 336 | 6106 | 0.19 | 1.83 |

**Table S3** Total levels of risk elements in soil

|  | As [mg∙kg^-1^] | Be [mg∙kg^-1^] | Cd [mg∙kg^-1^] | Co [mg∙kg^-1^] | Cr [mg∙kg^-1^] | Cu [mg∙kg^-1^] | Ni [mg∙kg^-1^] | Pb [mg∙kg^-1^] | V [mg∙kg^-1^] | Zn [mg∙kg^-1^] | Hg [mg∙kg^-1^] |
| --- | --- | --- | --- | --- | --- | --- | --- | --- | --- | --- | --- |
| 2021 | 13.6 | 1.25 | 0.43 | 16.1 | 67.4 | 30.2 | 45.5 | 31.0 | 52.7 | 113.0 | 0.140 |
| Long-term CZ monitoring (since 2019) | 9.13 | 0.95 | 0.24 | 11.6 | 38.8 | 20.1 | 25.4 | 25.6 | 40.5 | 70.4 | 0.065 |


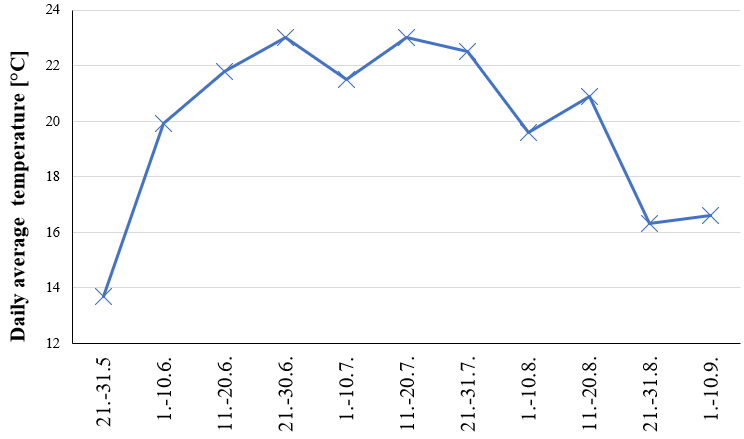

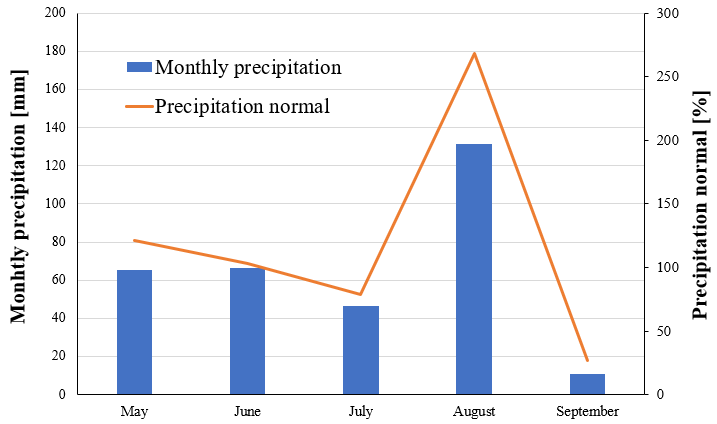


**Fig. S1** Trend of average daily temperatures and precipitation in the experimental field

**Sec**t**ion 3:** Statistical Analysis of the Influence of Extraction Parameters on Recovery Rate

**Table S4** Results of Tukey's HSD, Influence of Extraction Medium on RR of Enrofloxacin

| **Group 1** | **Group 2** | **MeanDiff** | **p-adj** | **Lower** | **Upper** | **Reject** |
| --- | --- | --- | --- | --- | --- | --- |
| EM1 | EM2 | 21.04 | 0.0417 | 1.351 | 40.729 | Tue |
| EM1 | EM3 | 40.86 | 0.0063 | 21.171 | 60.549 | True |
| EM1 | EM4 | 21.05 | 0.0615 | -1.6849 | 43.7849 | False |
| EM1 | EM5 | 4.28 | 0.8853 | -18.4549 | 27.0149 | False |
| EM1 | EM6 | 18.88 | 0.0818 | -3.8549 | 41.6149 | False |
| EM1 | EM7 | 21.36 | 0.04 | 1.671 | 41.049 | True |
| EM2 | EM3 | 19.82 | 0.0281 | 3.744 | 35.896 | True |
| EM2 | EM4 | 0.01 | 1.0 | -19.679 | 19.699 | False |
| EM2 | EM5 | -16.76 | 0.0768 | -36.449 | 2.929 | False |
| EM2 | EM6 | -2.16 | 0.9871 | -21.849 | 17.529 | False |
| EM2 | EM7 | 0.32 | 1.0 | -15.756 | 16.396 | False |
| EM3 | EM4 | -19.81 | 0.0492 | -39.499 | -0.121 | True |
| EM3 | EM5 | -36.58 | 0.0087 | -56.269 | -16.891 | True |
| EM3 | EM6 | -21.98 | 0.037 | -41.669 | -2.291 | True |
| EM3 | EM7 | -19.5 | 0.0294 | -35.576 | -3.424 | True |
| EM4 | EM5 | -16.77 | 0.1107 | -39.5049 | 5.9649 | False |
| EM4 | EM6 | -2.17 | 0.9934 | -24.9049 | 20.5649 | False |
| EM4 | EM7 | 0.31 | 1.0 | -19.379 | 19.999 | False |
| EM5 | EM6 | 14.6 | 0.1549 | -8.1349 | 37.3349 | False |
| EM5 | EM7 | 17.08 | 0.073 | -2.609 | 36.769 | False |
| EM6 | EM7 | 2.48 | 0.9758 | -17.209 | 22.169 | False |

**Table S5** Results of Tukey's HSD, Influence of Extraction Medium on RR of Ciprofloxacin

| **Group 1** | **Group 2** | **MeanDiff** | **p-adj** | **Lower** | **Upper** | **Reject** |
| --- | --- | --- | --- | --- | --- | --- |
| EM1 | EM2 | 23.58 | 0.0395 | 1.9563 | 45.2037 | True |
| EM1 | EM3 | 33.13 | 0.0151 | 11.5063 | 54.7537 | True |
| EM1 | EM4 | 14.94 | 0.1826 | -10.0289 | 39.9089 | False |
| EM1 | EM5 | 0.87 | 1.0 | -24.0989 | 25.8389 | False |
| EM1 | EM6 | 18.3 | 0.1124 | -6.6689 | 43.2689 | False |
| EM1 | EM7 | 1.271 | 0.9995 | -20.3527 | 22.8947 | False |
| EM2 | EM3 | 9.55 | 0.2284 | -8.1057 | 27.2057 | False |
| EM2 | EM4 | -8.64 | 0.4099 | -30.2637 | 12.9837 | False |
| EM2 | EM5 | -22.71 | 0.0438 | -44.3337 | -1.0863 | True |
| EM2 | EM6 | -5.28 | 0.7564 | -26.9037 | 16.3437 | False |
| EM2 | EM7 | -22.309 | 0.0262 | -39.9647 | -4.6533 | True |
| EM3 | EM4 | -18.19 | 0.0792 | -39.8137 | 3.4337 | False |
| EM3 | EM5 | -32.26 | 0.0163 | -53.8837 | -10.6363 | True |
| EM3 | EM6 | -14.83 | 0.1324 | -36.4537 | 6.7937 | False |
| EM3 | EM7 | -31.859 | 0.0094 | -49.5147 | -14.2033 | True |
| EM4 | EM5 | -14.07 | 0.2089 | -39.0389 | 10.8989 | False |
| EM4 | EM6 | 3.36 | 0.9678 | -21.6089 | 28.3289 | False |
| EM4 | EM7 | -13.669 | 0.1608 | -35.2927 | 7.9547 | False |
| EM5 | EM6 | 17.43 | 0.1268 | -7.5389 | 42.3989 | False |
| EM5 | EM7 | 0.401 | 1.0 | -21.2227 | 22.0247 | False |
| EM6 | EM7 | -17.029 | 0.0938 | -38.6527 | 4.5947 | False |

**Table S6** Results of Tukey's HSD, Influence of Extraction Temperature on RR of Enrofloxacin

| **Group 1** | **Group 2** | **MeanDiff** | **p-adj** | **Lower** | **Upper** | **Reject** |
| --- | --- | --- | --- | --- | --- | --- |
| 25 °C | 35 °C | 6.82 | 0.044 | 0.2642 | 13.3758 | True |
| 25 °C | 45 °C | 3.07 | 0.3522 | -3.4858 | 9.6258 | False |
| 25 °C | 55 °C | 2.68 | 0.4424 | -3.8758 | 9.2358 | False |
| 35 °C | 45 °C | -3.75 | 0.2344 | -10.3058 | 2.8058 | False |
| 35 °C | 55 °C | -4.14 | 0.1858 | -10.6958 | 2.4158 | False |
| 45 °C | 55 °C | -0.39 | 0.9942 | -6.9458 | 6.1658 | False |

**Table S7** Results of Tukey's HSD, Influence of Extraction Temperature on RR of Ciprofloxacin

| **Group 1** | **Group 2** | **MeanDiff** | **p-adj** | **Lower** | **Upper** | **Reject** |
| --- | --- | --- | --- | --- | --- | --- |
| 25 °C | 35 °C | 34.91 | 0.0036 | 18.2252 | 51.5948 | True |
| 25 °C | 45 °C | 30.24 | 0.0062 | 13.5552 | 46.9248 | True |
| 25 °C | 55 °C | 30.08 | 0.0063 | 13.3952 | 46.7648 | True |
| 35 °C | 45 °C | -4.67 | 0.6887 | -21.3548 | 12.0148 | False |
| 35 °C | 55 °C | -4.83 | 0.669 | -21.5148 | 11.8548 | False |
| 45 °C | 55 °C | -0.16 | 1 | -16.8448 | 16.5248 | False |

**Section 4:** Correlation between Cumulative Rainfall and Fluoroquinolone concentrations in the soil

Fig. S2B

Fig. S2A


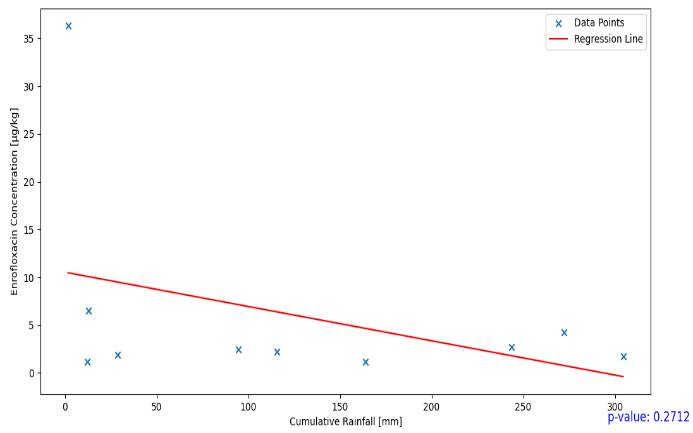

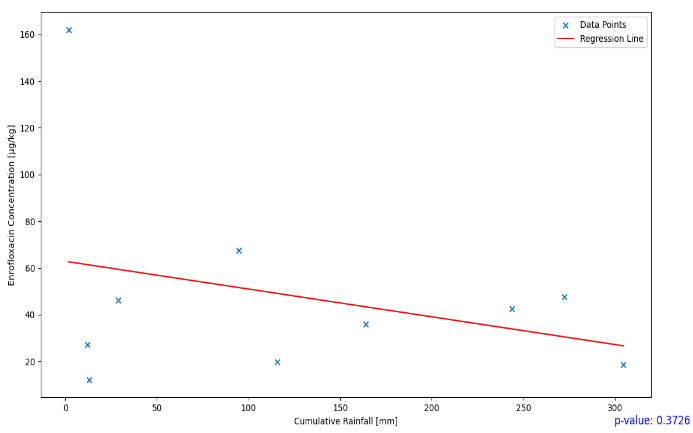


Fig. S2D

Fig. S2C


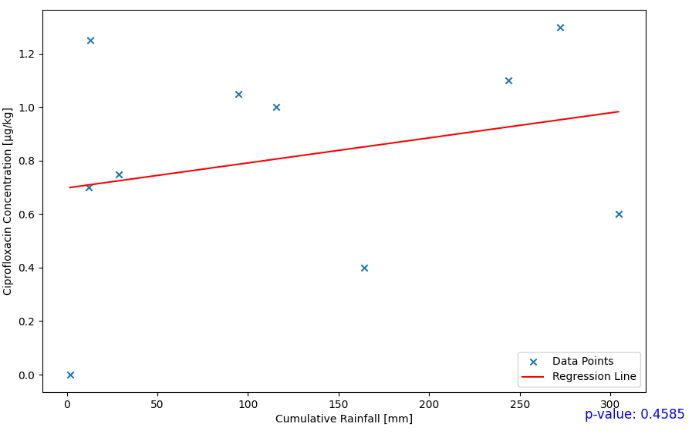

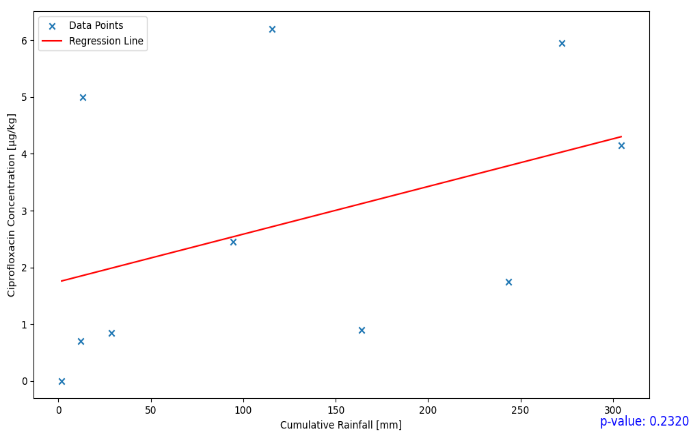


**Fig. S2** Correlation between Cumulative Rainfall and Fluoroquinolone concentrations in soil; 2A) Enrofloxacin, non-spiked poultry litter; 2B) Enrofloxacin, spiked poultry litter; 2C) Ciprofloxacin, non spiked poultry litter; 2D) Ciprofloxacin, spiked poultry litter**Section 5:** Calculated RQ values for ENR and CIP

**Table S8** Calculated RQ values for ENR and CIP

| **Number of days since poultry litter incorporation into soil** | **RQ ENR [-]** | **RQ**  **MIC-ENR [-]** | **RQ CIP [-]** | **RQ**  **MIC-CIP [-]** |
| --- | --- | --- | --- | --- |
| 0 | 0.04 | 1.41 | 0.00 | 0.00 |
| 7 | 0.00 | 0.05 | 0.00 | 0.02 |
| 14 | 0.01 | 0.25 | 0.00 | 0.04 |
| 28 | 0.00 | 0.07 | 0.00 | 0.02 |
| 42 | 0.00 | 0.10 | 0.00 | 0.03 |
| 56 | 0.00 | 0.09 | 0.00 | 0.03 |
| 70 | 0.00 | 0.04 | 0.00 | 0.01 |
| 84 | 0.00 | 0.11 | 0.00 | 0.03 |
| 101 | 0.01 | 0.16 | 0.01 | 0.04 |
| 115 | 0.00 | 0.07 | 0.00 | 0.02 |

**REFERENCES**

Boxall, A. B. A.; Johnson, P.; Smith, E. J.; Sinclair, C. J.; Stutt, E.; Levy, L. S. Uptake of Veterinary Medicines from Soils into Plants. Journal of Agricultural and Food Chemistry 2006, 54 (6), 2288–2297. <https://doi.org/10.1021/jf053041t>.

Sanford, J. C.; Mackie, R. I.; Koike, S.; Krapac, I. G.; Lin, Y.-F.; Yannarell, A. C.; Maxwell, S.; Aminov, R. I. Fate and Transport of Antibiotic Residues and Antibiotic Resistance Genes Following Land Application of Manure Waste. Journal of Environmental Quality 2009, 38 (3), 1086–1108. <https://doi.org/10.2134/jeq2008.0128>.
